# Efficacy and safety of radiofrequency ablation (RFA) for recurrent metastatic thyroid carcinoma to the lateral neck: a systematic review

**DOI:** 10.1007/s00405-025-09563-x

**Published:** 2025-07-12

**Authors:** Carlos Galan, Devanshu Kwatra, Ananth Vijendren, Anant Patel, Kanchana Rajaguru, Panagiotis A. Dimitriadis, George Mochloulis

**Affiliations:** 1 ENT Department, Clinica Universidad de Navarra, Madrid, Spain; 2 Lister Thyroid Centre, East and North Hertfordshire Teaching NHS Trust, Stevenage, UK; 3 Department of Radiology, Lister Hospital, East and North Hertfordshire Teaching NHS Trust, Stevenage, UK

## Abstract

**Aim:**

To evaluate the efficacy and safety of radiofrequency ablation (RFA) for lateral cervical recurrence of metastatic thyroid carcinoma compared to revision surgery (after a primary thyroidectomy and selective neck dissection) and RFA in the central compartment or thyroid bed. This review focuses on studies that differentiate between lateral and central neck RFA, as lesion location might influence outcomes and complication rates.

**Methods:**

A systematic review was conducted using the PRISMA guidelines, looking at articles that address management of thyroid cancer recurrence in the lateral neck. Data on tumor volume reduction, thyroglobulin (Tg) levels, recurrence rates, and complications were analyzed.

**Results:**

Eight studies were include in this review. Across 252 patients treated with RFA, complete nodule disappearance was reported in up to 97% of cases, with therapeutic success rates ranging from 92 to 100%. Volume reduction rates (VRR) reached 93–100% at 24–60 months. Thyroglobulin levels consistently decreased post-treatment, with reductions from baseline values as high as 37 ng/mL to < 1 ng/mL. Recurrence-free survival was 90.8–96% at 1–3 years, and up to 94.5% at 6 years in long-term follow-up. Radiofrequency ablation demonstrated a favorable safety profile: transient voice changes occurred in 3.7–8.6% of patients, mainly in central lesions, with no reported cases of hypocalcemia. Surgery showed higher complication rates, including hypocalcemia (8.7–30%) and permanent voice changes. Comparative studies showed that while surgery may offer more frequent biochemical remission, RFA remains effective with fewer complications.

**Conclusion:**

RFA is an effective, minimally invasive alternative for lateral cervical recurrence of thyroid cancer. Its high efficacy and superior safety profile make it a valuable option, particularly for patients avoiding revision surgery. Differentiating lateral and central RFA is important, as both show comparable efficacy, though central RFA has a higher complication risk.

## Introduction

Thyroid cancer is the most common endocrine malignancy [1] and although it generally has an excellent prognosis, loco-regional recurrences occur in a subset of patients, particularly within the cervical lymph nodes [2]. Traditional management of cervical recurrences has involved repeat surgical resection; however, reoperations carry significant risks, including recurrent laryngeal nerve injury and hypoparathyroidism [3].

Radiofrequency ablation (RFA) has emerged as a minimally invasive alternative for managing loco-regional recurrences [4, 5] offering the potential for effective tumor control with a lower complication profile. While numerous studies have explored the role of RFA in thyroid cancer [6, 7] few have clearly distinguished outcomes based on the location of recurrence, specifically between the lateral and central compartments. Given the anatomical complexity and heightened risk of complications associated with central compartment interventions, evaluating outcomes by anatomical site is essential. This systematic review focuses specifically on the efficacy and safety of RFA for metastatic thyroid carcinoma to the lateral neck, comparing these outcomes to those of conventional surgical treatments and RFA in the central compartment.

## Methods

A systematic review was conducted following PRISMA guidelines [8]. Comprehensive searches were performed across PubMed, Embase, and the Cochrane Library up to December 2025. Search terms included combinations of “thyroid cancer and radiofrequency ablation and lateral neck recurrence and cervical lymph node metastases.”

Inclusion criteria:

- Studies involving patients with metastatic thyroid carcinoma undergoing RFA for lateral neck recurrences.
- Studies where either outcomes, complications, or both were clearly reported separately for lateral versus central or thyroid bed lesions.
- Studies reporting at least two of the following outcomes: tumor volume reduction, Tg levels, recurrence rates, and complication rates.

Exclusion criteria:

- Studies focusing exclusively on thyroid bed or central compartment lesions.
- Case reports, reviews, and non-English language articles.

**Fig. 1:**
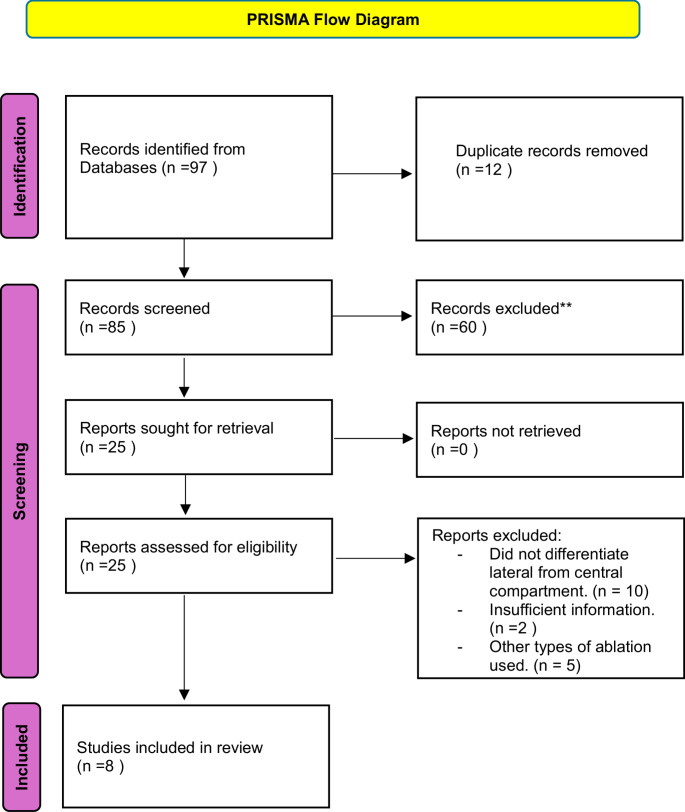
PRISMA 2020 flow diagram for new systematic reviews which included searches of databases and registers only

Data were extracted on sample size, patient demographics, tumor location, tumor volume reduction rates, serum Tg changes, recurrence-free survival rates, and complication rates. All efficacy outcomes and tables reflect data from studies with a predominance of lateral lesions; however, some aggregated quantitative outcomes may include a minority of central lesions when separate data were not available. However, complication data were analyzed separately in all cases (Fig. 1).

## Results

From an initial pool of 97 articles, 8 studies were selected that met the inclusion criteria. They included 252 patients with 344 with either lateral, central or both neck recurrences of metastatic thyroid carcinoma treated with radiofrequency ablation (RFA). The average number of nodules treated per patient was 1.33, with a range from 1 to 3. Most studies focused on lateral compartment lesions, although some also included central compartment. Follow-up periods ranged from 12 to 80 months.

Therapeutic success was consistently high across all studies. Complete disappearance of treated nodules was reported in up to 97% of cases [4] while Yang Z et al. observed 100% volume reduction over long-term follow-up [7]. Volume reduction rates (VRR) were similarly favorable, ranging from 93 to 100% in studies by Yang Z [7] Chung SR [8]and Yang G [9] with Baek et al. reporting a 93% VRR at just 12 months [6].

In terms of biochemical response, all studies that measured serum Tg showed significant post-RFA decreases. The highest pre-treatment Tg level (37 ng/mL) was reported by Monchik, which fell to 1.9 ng/mL post-treatment [5]. Baek observed a decrease from 8.1 to 2.2 ng/mL^6^, while Yang G reported levels falling from 10.2 to 1.1 ng/mL [10]. Yang Z noted a median drop from 1.48 to 0.00 ng/mL^7^.

Recurrence rates post-RFA varied slightly across cohorts. Kim JH et al. observed an 11.5% recurrence rate at 32 months [4] whereas Yang Z reported 28% new recurrences that were all successfully managed with additional RFA [7]. Long-term recurrence-free survival (RFS) ranged from 90.8 to 96% at 1–3 years and from 89.5 to 94.5% at 6 years, with these outcomes reported in larger cohorts such as Chung [8] and Choi [11].

Complication rates were uniformly low. Transient voice changes occurred in 3.7–8.6% of RFA cases, most frequently in central neck procedures. Permanent hoarseness was rare (< 1%). Importantly, no cases of hypocalcemia were reported following RFA, contrasting with the 8.7–30% hypocalcemia rate reported in surgical cohorts (Table 1).

**Table 1:** Summary of outcomes following RFA. This table includes tumor volume reduction, thyroglobulin reduction and complications to assess the efficacy and safety of the procedure

Author (Year)	Patients	Treated location	Follow-up (months)	VRR (%)	Tg reduction (%)	Main complications
Ji-hoon Kim	27	17 lateral, 10 central	32	97.1	46.43	3.7% transient voice change
Monchik	16	13 lateral, 3 central	38	N/R	94.86	1 Transient voice change (central lesion)
Jung Hwan Baek	10	11 lateral, 1 central	12	93	72.84	1 Transient voice change (surgical bed)
Zhen Yang	32	49 lateral, 9 central	60	100	100	No complications
Sun Jin Lee	35	9 lateral, 26 central	30	86.78	84.22	Transient voice changes (only in central)
Yang Guang	33	45 lateral, 9 central	12–24	94.9	89.22	No complications
Yangsean Choi	70	32 lateral, 64 central	36	N/R	95.6	8.3% transient voice change
Sae Rom Chung	29	23 lateral, 23 central	80	99.5	70.59	3 transient voice changes

While surgery showed a higher rate of biochemical remission in certain cases, this must be weighed against the greater complication burden associated with surgical reintervention. In Kim JH et al., biochemical remission was achieved in 78.6% of surgical patients versus 28.6% in those treated with RFA, though the larger Tg decline observed with surgery (10.14 ± 19.15 ng/mL vs. 2.67 ± 4.09 ng/mL) did not reach statistical significance [4]. Additionally, a greater proportion of RFA patients had post-treatment Tg ≥ 1 ng/mL (24% vs. 7.3%), yet Choi et al. demonstrated that long-term recurrence-free survival remained comparable between the two groups, with RFA showing a markedly lower incidence of major complications [11] (Table 2).

**Table 2:** Complication rates for RFA versus revision surgery, highlighting differences in safety profiles

Complication	RFA (%)	Revision surgery (%)
Transient vocal cord palsy	3.7-8.6%	18%
Transient hypocalcemia	0%	8.7-30%
Permanent hypocalcemia	0%	8.7-26%
Permanent vocal cord palsy	<1% (very rare)	> 5%
Other complications	Negligible	Frequent

## Discussion

The findings of this systematic review highlight RFA as an effective and safe alternative to repeat surgery for managing lateral cervical recurrences of metastatic thyroid carcinoma. The high therapeutic success rates, substantial tumor volume reductions, and significant decreases in serum thyroglobulin levels reaffirm the efficacy of RFA [4–7]. Moreover, recurrence-free survival rates post-RFA are comparable to those following surgical reintervention [9, 11].

Importantly, RFA demonstrates a superior safety profile, particularly regarding the absence of permanent hypocalcemia and a lower incidence of voice changes. Complications were more frequently reported in central compartment procedures, likely reflecting the increased anatomical complexity and proximity to critical structures such as the recurrent laryngeal nerve [11, 12]. These findings reinforce the clinical importance of distinguishing between lateral and central neck interventions. In particular, the consistent association of central RFA with vocal cord dysfunction underscores the need for anatomical precision in risk assessment.

This review highlights the importance of future studies clearly reporting complications according to compartment, as this will help clarify the real morbidity attributable to central interventions and allow refinement of treatment selection criteria.

Although percutaneous ethanol injection (PEI) has historically been used as a minimally invasive treatment option, particularly for small or difficult-to-access recurrent thyroid lesions [13] its role has become more limited in recent years. RFA has emerged as the preferred modality for treating solid and mixed thyroid nodules due to its higher efficacy, lower recurrence rates, and reduced need for multiple treatment sessions [14]. Current clinical guidelines now recommend RFA as the first-line ablative technique for symptomatic benign solid nodules and selected cases of loco-regional recurrence of differentiated thyroid carcinoma, while PEI is generally reserved for predominantly cystic lesions [14, 15].

This review underscores the necessity of meticulous patient selection and technical expertise to optimize clinical outcomes. Limitations of the included studies, notably small sample sizes, retrospective designs, and heterogeneous follow-up protocols, must be considered. Future randomized and well-organized studies comparing the outcomes of RFA and surgery are essential to validate these findings and establish standardized protocols for RFA in the management of recurrent thyroid carcinoma.

RFA should be considered in selected patients as an alternative to surgery, which remains the first-line and potentially curative treatment. It should be considered for patients with contraindications to surgery, higher surgical risk due to previous operations, or those who decline reoperation. In these cases, the goal of RFA is to control the disease rather than to achieve complete biochemical remission. Although thyroglobulin levels may not decrease as much as with surgery, available evidence suggests that survival outcomes are similar. The slower radiological disappearance of lesions and the anxiety this may cause should be clearly explained to patients before making a decision.

## Strengths and limitations

Strengths of this systematic review include a focused analysis specifically on lateral neck recurrences, differentiation between lateral and central compartment outcomes, and comprehensive inclusion of relevant clinical endpoints such as tumor volume reduction, biochemical response, and complication rates

However, several limitations must be acknowledged. First, the number of studies included was relatively small, and most were retrospective in nature, introducing potential bias. Second, heterogeneity among studies in terms of RFA technique, energy settings, and follow-up duration limits direct comparability. Third, although we focused on studies with predominantly lateral neck lesions and extracted data specific to lateral recurrences, in some cases the efficacy outcomes may include a minority of central lesions when separate data were not available. Finally, publication bias may exist, as studies with positive outcomes are more likely to be published

## Conclusion

Radiofrequency ablation represents an effective and safe therapeutic option for patients with lateral cervical recurrence of metastatic thyroid carcinoma. With high success rates, substantial tumor reduction, and a favorable safety profile compared to repeat surgery, RFA offers a minimally invasive alternative that preserves patient quality of life. Although complications are more frequent when treating central compartment lesions, overall efficacy appears comparable between lateral and central RFA

This review underscores the importance of conducting well-designed, randomized studies comparing the outcomes of RFA and surgery, ideally incorporating cost-effectiveness analyses. Moreover, future research should consistently report complications according to anatomical location, particularly for central compartment procedures, given their stronger association with recurrent laryngeal nerve injury. This distinction is essential for refining patient selection, improving procedural planning, and enhancing safety outcomes in clinical practice

## Data Availability

The data presented in this review were obtained from previously published studies, all of which adhered to the ethical standards required by the respective institutional and national guidelines.
