# Correction: Efficacy and safety of radiofrequency ablation (RFA) for recurrent metastatic thyroid carcinoma to the lateral neck: a systematic review

**DOI:** 10.1007/s00405-025-09639-8

**Published:** 2025-08-29

**Authors:** Carlos Galan, Devanshu Kwatra, Ananth Vijendren, Anant Patel, Kanchana Rajaguru, Panagiotis A. Dimitriadis, George Mochloulis

**Affiliations:** 1 ENT Department, Clinica Universidad de Navarra, Madrid, Spain; 2 Lister Thyroid Centre, East and North Hertfordshire Teaching NHS Trust, Stevenage, UK; 3 Department of Radiology, Lister Hospital, East and North Hertfordshire Teaching NHS Trust, Stevenage, UK

The original article can be found online at 10.1007/s00405-025-09563-x.

**Correction: European Archives of Oto-Rhino-Laryngology**

10.1007/s00405-025-09563-x

In this article, the author's name - Carlos Galan was inadvertently included twice in the author group. The correct author group should be as in this publication. The original article has been corrected.

